# Impact of age, sex, race, and regionality on major clinical outcomes of COVID-19 in hospitalized patients in the United States

**DOI:** 10.1186/s12879-022-07611-z

**Published:** 2022-07-29

**Authors:** Suneha S. Sundaram, Stephanie Melquist, Pankush Kalgotra, Sachin Srinivasan, Sravanthi Parasa, Madhav Desai, Prateek Sharma

**Affiliations:** 1 Department of Internal Medicine, Robert Wood Johnson Barnabas Health, Rahway, NJ USA; 2 Department of Gastroenterology and Hepatology, Veterans Affairs Medical Center, 4801 Linwood Blvd, Kansas City, MO 64128 USA; 3 Department of Internal Medicine, Sanford Health, Fargo, ND USA; 4 Department of Internal Medicine, University of North Dakota School of Medicine, Grand Forks, ND USA; 5 Department of Systems and Technology, Harbert College of Business, Auburn University, Auburn, AL USA; 6 Division of Gastroenterology, Hepatology and Motility, Department of Internal Medicine, University of Kansas School of Medicine, Kansas City, KS USA; 7 Department of Gastroenterology, Swedish Medical Center, Seattle, WA USA

**Keywords:** COVID-19, SARS-CoV-2, Racial disparities, Risk factors, Mortality, ARDS, Mechanical ventilation, Sepsis, Outcomes

## Abstract

**Background:**

The COVID-19 pandemic has affected all people across the globe. Regional and community differences in timing and severity of surges throughout the pandemic can provide insight into risk factors for worse outcomes in those hospitalized with COVID-19.

**Methods:**

The study cohort was derived from the Cerner Real World Data (CRWD) COVID-19 Database made up of hospitalized patients with proven infection from December 1, 2019 through November 30, 2020. Baseline demographic information, comorbidities, and hospital characteristics were obtained. We performed multivariate analysis to determine if age, race, comorbidity and regionality were predictors for mortality, ARDS, mechanical ventilation or sepsis hospitalized patients with COVID-19.

**Results:**

Of 100,902 hospitalized COVID-19 patients included in the analysis (median age 52 years, IQR 36–67; 50.7% female), COVID-19 case fatality rate was 8.5% with majority of deaths in those ≥ 65 years (70.8%). In multivariate analysis, age ≥ 65 years, male gender and higher Charlson Comorbidity Index (CCI) were independent risk factors for mortality and ARDS. Those identifying as non-Black or non-White race have a marginally higher risk for mortality (OR 1.101, CI 1.032–1.174) and greater risk of ARDS (OR 1.44, CI 1.334–1.554) when compared to those who identify as White. The risk of mortality or ARDS was similar for Blacks as Whites. Multivariate analysis found higher mortality risk in the Northeast (OR 1.299, CI 1.22–1.29) and West (OR 1.26, CI 1.18–1.34). Larger hospitals also had an increased risk of mortality, greatest in hospitals with 500–999 beds (OR 1.67, CI 1.43–1.95).

**Conclusion:**

Advanced age, male sex and a higher CCI predicted worse outcomes in hospitalized COVID-19 patients. In multivariate analysis, worse outcomes were identified in small minority populations, however there was no difference in study outcomes between those who identify as Black or White.

**Supplementary Information:**

The online version contains supplementary material available at 10.1186/s12879-022-07611-z.

## Background

COVID-19 caused by the severe acute respiratory syndrome coronavirus 2 (SARS-CoV-2) continues to wreak havoc 2 years after its discovery. Some communities have suffered disproportionately more than others. Based on early data, increasing age, male sex, and certain comorbidities were recognized as risk factors for poor outcomes of COVID-19 [1–4]. Non-white race has been identified as a risk factor for hospitalization and poor outcomes [5–8]. As data has amassed, we are able to investigate trends for morbidity and mortality and define risk factors for hospitalization due to COVID-19 and make necessary changes to protect these groups with evidence-based knowledge.

This large, US based retrospective study describes the demographics and comorbidities of hospitalized patients with COVID-19 infection from the CRWD, COVID-19 database. We compare hospital specific and patient characteristics that may predict poor outcomes of COVID-19 disease from the first year of the pandemic.

## Methods

### Study design and population

The study cohort was derived from the CRWD COVID-19 Database. This national server of de-identified patient data was created by Cerner for the purpose of academic research. Data was extracted from the Cerner electronic health record (EHR) from 87 hospital systems who consented for inclusion in this database. Cerner de-identified all data in compliance with the Health Insurance Portability and Accountability Act. Access is granted under a data use agreement to organizations and individuals after approval of a submitted research proposal. Encounters may include pharmacy, clinical and microbiology laboratory, admission and billing information from affiliated patient care locations. All admissions, medication orders and dispensing, laboratory orders and specimens are date and time-stamped, providing a temporal relationship between treatment patterns and clinical information. Patients with at least one encounter (emergency department or inpatient) with a diagnosis code associated with COVID-19 exposure or infection, or an emergency department or inpatient encounter with a positive result for a COVID-19 laboratory test were eligible for inclusion in the CRWD COVID-19 Database. Only diagnoses and laboratory results from encounters with a service date of 12/1/2019 through 11/30/2020 were included. Longitudinal data was included from all encounters for each patient (labeled by a unique identification number) from January 2015 to account for variables of interest in the medical history. All available data was utilized to define our study population and no patient files were excluded based on demographic data or other clinical reasoning. Inclusion criteria was defined as (1) any patient with a minimum of one emergency department visit or hospitalization with a diagnosis code associated to COVID-19 infection, or (2) an ER or hospital admission with a positive result for a COVID-19 test from December 1, 2019 through November 30, 2020. Their data was then linked to visits coded as COVID-19 visits. Based on the definitions of different diseases and complications defined in Additional file 1: Table S2, we created multiple binary variables representing the presence or absence of each outcome in each patient. The dataset about visits was merged with diagnoses codes. Full details of the CRWD COVID-19 Database are available upon request by emailing: learninghealthnetwork@cerner.com. The study was exempt from review under the minimal risk protocol from Providence St. Joseph Hospital IRB, Seattle, WA, USA.

### Definition of variables and statistical analysis

From this cohort of hospitalized COVID-19 patients, we used International Classification of Diseases, Clinical Modification (ICD-9-CM), Ninth and Tenth Revision (ICD-9-CM and ICD-10-CM) diagnosis codes to identify any patient with sepsis, acute respiratory distress syndrome (ARDS), need of mechanical ventilation or death. A complete list of ICD codes for outcomes of interest and comorbidities are listed in Additional file 1: Table S1. These codes were queried for the encounters coinciding with the first positive COVID-19 lab result or diagnosis code only. If a patient was diagnosed with COVID-19 on a single encounter and was subsequently diagnosed with a severe outcome at a future date, these encounters were not included.

Data collected includes patient demographics, comorbidities, diagnoses during the hospital course, treatments, outcomes and hospital characteristics. Race was defined from fixed categories given in the CRWD database. White, Black and “other” races were included. The category “other” was defined as any patient who identified as Asian or “other”. Regions of hospital admission were defined as Northeast, South, West and Midwest collected from fixed categories in Cerner database defined by hospital area code. Hospital size was pre-determined by number of hospital beds.

Poor outcomes were identified by ICD-9 and ICD-10 diagnosis codes and include mechanical ventilation (defined as any patient with a code pertaining to dependence on a respiratory ventilator), ARDS (defined as any patient with this specific diagnosis code) and sepsis (identified for any patient with a code for sepsis with or without a specified organism).

All calculations for poor outcomes were reported as “case rates”, calculated as COVID-19 related number of instances for any given poor outcome divided by the incident cases of COVID-19 hospitalized patients. The rationale for this was mortality rate is defined as the number of deaths divided by the size of the population from which the deaths occurred. The total number of hospital admissions and all COVID-19 infection rates (inpatient and outpatient) were not available in CRWD, therefore accurate mortality rates could not be assessed. All patients were known to have COVID-19, thus case fatality rate was defined as the number of COVID-19 specific deaths divided by the total number of incident COVID-19 cases [9]. We defined our cohort by incident cases and used the logistics of case fatality rate to translate this to all outcomes.

Bivariate logistic regression analysis was used to compare patient demographics, hospital characteristics, and comorbidities to disease outcomes. Multivariate logistic regression model with all variables was performed to estimate the adjusted odds risk of each outcome of interest. The threshold for statistical significance was set to a *p* value of 0.05 for all analyses. For multivariate logistic regression analysis, Odds Ratios (OR) and 95% Confidence Intervals (CI) determined by likelihood ratio chi-square analysis were used to determine statistical significance. Statistical analysis was performed using Python 3.10 (Create Space) and SAS 9.4 (SAS Institute Inc.) programming languages.

### Study outcomes

The outcomes of interest were rates of severe complications of COVID-19 in hospitalized patients including death, sepsis, mechanical ventilation and ARDS. These outcomes were compared by demographics including age, sex, race and Charlson Comorbidity Index (CCI), Hospital characteristics included US region and bed size.

## Results

### Demographics

Our cohort is comprised of 100,902 patients (median age 52 years, IQR 36–67; 50.7% female), with mean length of stay of 6.83 days (SD 15.73). By age, 27.1% patients were ≥ 65 years, of which 9% were > 80 years old. The majority (58.1%) of patients in the cohort were White, 18.4% were Black, and 23.5% patients identified as “other” races. The mean CCI was 1.27 (SD ± 2.02) with 55.3% patients with CCI = 0. There were 35.7% with a score of 1–4, and 8.9% with scores ≥ 5. The most common comorbidities identified were hypertension (31%) and diabetes mellitus (25.4%). Forty percent of patients were admitted to hospitals in the South, 31% in the West, 20% in the Northeast and 8% in the Midwest. Over half (67%) of patients were admitted to very large hospitals (> 1000 beds), 20% from large hospitals (500–999 beds) and 10% from hospitals with 200–499 beds.

The case fatality rate was 8.5% (p < 0.00001). Ten percent of patients were diagnosed with sepsis (p < 0.00001), 4.1% with ARDS (p < 0.00001) and 3.1% required mechanical ventilation (p < 0.00001). Complete demographics, hospital characteristics, comorbidity data and outcomes are summarized in Table 1. All proportions met statistical significance.

**Table 1:** Baseline demographics, comorbidities and outcomes

Hospital and ER admissions		
N = 100,902		
	n	Proportion
Age (y)		
Mean (SD)	52.39 (19.32)	
Median (IQR)	52 (67–36)	
< 17 year	8238	8.20%
18–34	20,822	20.60%
35–49	20,661	20.50%
50–64	23,821	23.60%
65–79	18,316	18.20%
≥ 80	9044	9.00%
Sex		
Men	49,331	48.90%
Women	51,172	50.70%
Race		
White	58,655	58.10%
Black	18,538	18.40%
Other	23,709	23.50%
Ethnicity		
Hispanic	43,266	42.9%
Non-hispanic	53,343	52.90%
Other or unknown	4293	4.30%
Hospital region		
Midwest	8309	8.20%
Northeast	20,827	20.60%
South	40,448	40.10%
West	31,318	31.00%
Hospital beds, no		
1–99	1024	1.00%
100–199	825	0.80%
200–299	2751	2.70%
300–499	7983	7.90%
500–999	20,972	20.80%
> 1000	67,347	66.70%
CCI		
Charlson Comorbidity Index mean (SD)	1.217 (2.02)	
0	55,848	55.30%
1–4	36,072	35.70%
≥ 5	8982	8.90%
Charlson comorbidities		
Myocardial infarction	4060	4.00%
Congestive heart Failure	8269	8.20%
Peripheral vascular disease	2995	3.00%
Cerebrovascular disease	3240	3.20%
Dementia	5597	5.50%
COPD	13,865	13.70%
Rheumatological disease	1458	1.40%
Peptic ulcer disease	475	0.50%
Mild Liver disease	3517	3.50%
DM with no complications	23,411	23.20%
DM with chronic complications	7842	7.80%
Hemiplegia/ Paraplegia	787	0.80%
Kidney disease	10,345	10.30%
Any malignancy, including leukemia or lymphoma	2389	2.40%
Moderate or severe liver disease	711	0.70%
Metastatic solid tumor	605	0.60%
AIDS/ HIV	428	0.40%
Hypertension	31,258	31.00%
Hyperlipidemia	17,549	17.40%
Concomitant GI conditions		
Acute liver failure	444	0.40%
Chronic GERD	7541	7.50%
GI malignancy, ALL	376	0.40%
GI malignancy, esoph/ stomach/colon/liver	265	0.30%
IBD (UC + chrons)	316	0.30%
Celiac disease	36	0.00%
Length of stay		
Mean (SD)	6.83 days (15.73)	
Median (IQR)	2 (8)	
Mortality		
Total in hospital mortality	8574	8.5%
Severe complications		
ARDS	4135	4.1%
Sepsis	16,145	16.0%
AKI	14,782	14.6%
VTE	1559	1.5%
Chronic ischemic heart disease	8574	8.4%
Respiratory failure	27,829	27.6%
Use of mechanical ventilation	3177	3.1%
GI specific complications		
GI bleeding (GI Hem)	2000	1.98%
Intestinal ischemia	37	*
Pancreatitis	554	*
Acute liver injury	444	*
Dysphagia	2899	2.87%
Pseudo obstruction/Ogilvie	13	*

*Proportion less than 1% (NB: All proportions met statistical significance with *p* < 0.0001)

### Impact of age

The majority of patients who died were ≥ 65-year-old (70.8%) with a case fatality rate of 22.2%. Specifically, the case fatality rate for patients > 80 years old was 30.4% and 18.1% for patients 65–79 years old (p < 0.00001). Case fatality rate for those younger than 65 was less, with steady decline with younger ages (Table 2). On multivariate analysis, increased age was an independent risk factor for mortality with odds risk approximately doubling with each subsequent age group (Table 3).

**Table 2:** Case rates of severe outcomes by demographics and hospital characteristics

	Hospitalized/ ER		Mortality		ARDS		Invasive ventilation		Sepsis	
	100,902		8574		4135		3177		16,145	
	n	Case rate	n	Case rate	n	Case rate	n	Case rate	n	Case rate
Age (year)										
Mean (SD)	52.39 (19.32)									
Median (IQR)	52 (67–36)									
< 18	8238	8.2%	16	0.19%	19	0.23%	32	0.39%	281	3.41%
18–34	20,822	20.6%	147	0.71%	215	1.03%	83	0.40%	1134	5.45%
35–49	20,661	20.5%	496	2.40%	641	3.10%	332	1.61%	2454	11.88%
50–64	23,821	23.6%	1846	7.75%	1435	6.02%	916	3.85%	4590	19.27%
65–79	18,316	18.2%	3320	18.13%	1451	7.92%	1278	6.98%	5068	27.67%
≥ 80	9044	9.0%	2749	30.40%	374	4.14%	536	5.93%	2618	28.95%
Sex										
Men	49,331	48.9%	4986	10.11%	2504	5.08%	1657	3.36%	9135	18.52%
Women	51,172	50.7%	3545	6.93%	1614	3.15%	1503	2.94%	6928	13.54%
Race										
White	58,655	58.1%	5382	9.18%	2306	3.93%	2080	3.55%	9825	16.75%
Black	18,538	18.4%	1480	7.98%	610	3.29%	478	2.58%	2650	14.29%
Other	23,709	23.5%	1712	7.22%	1219	5.14%	619	2.61%	3670	15.48%
Ethnicity										
Hispanic	43,266	42.9%	3002	6.94%	1894	4.38%	1102	2.55%	6659	15.39%
Non-hispanic	53,343	52.9%	5221	9.79%	2018	3.78%	1958	3.67%	8761	16.42%
Other or unknown	4293	4.3%	351	8.18%	223	5.19%	117	2.73%	725	16.89%
Hospital region										
Midwest	8309	8.2%	654	7.87%	286	3.44%	192	2.31%	655	7.88%
Northeast	20,827	20.6%	2204	10.58%	815	3.91%	919	4.41%	4194	20.14%
South	40,448	40.1%	3063	7.57%	1182	2.92%	967	2.39%	5429	13.42%
West	31,318	31.0%	2653	8.47%	1852	5.91%	1099	3.51%	5867	18.73%
Hospital size (n)										
1–99	1024	1.0%	64	6.25%	16	1.56%	33	3.22%	73	7.13%
100–199	825	0.8%	45	5.45%	49	5.94%	8	0.97%	113	13.70%
200–299	2751	2.7%	104	3.78%	106	3.85%	97	3.53%	153	5.56%
300–499	7983	7.9%	540	6.76%	202	2.53%	208	2.61%	731	9.16%
500–999	20,972	20.8%	2170	10.35%	868	4.14%	613	2.92%	2968	14.15%
> 1000	67,347	66.7%	5651	8.39%	2894	4.30%	2218	3.29%	12,107	17.98%
Comorbidity										
Charlson Comorbidity Index mean (SD)	1.217 (2.02)									
0	55,848	55.3%	1209	2.16%	801	1.43%	391	0.70%	3526	6.31%
1–4	36,072	35.7%	4711	13.06%	2467	6.84%	1817	5.04%	8779	24.34%
≥ 5	8982	8.9%	2654	29.55%	867	9.65%	969	10.79%	3840	42.75%

**Table 3:** Multivariate effect of race, sex, age, comorbidity and region on outcomes in COVID-19 hospitalizations

Effect	Mortality			ARDS			Mechanical ventilation			Sepsis		
	Adjusted OR	95% Wald confidence interval		Adjusted OR	95% Wald confidence interval		Adjusted OR	95% Wald confidence interval		Adjusted OR	95% Wald confidence interval	
Race*												
Black	0.958	0.895	1.025	0.93	0.84	1.02	**0.75**	**0.68**	**0.84**	**0.90**	**0.86**	**0.95**
All others	**1.10**	**1.03**	**1.17**	**1.44**	**1.33**	**1.55**	**0.62**	**0.74**	**0.90**	1.05	1.00	1.10
Sex**												
Male	**1.49**	**1.42**	**1.57**	**1.51**	**1.41**	**1.61**	1.02	0.95	1.09	**1.38**	**1.33**	**1.43**
Age***												
Age 18–34	**3.12**	**1.86**	**5.23**	**3.62**	**2.26**	**5.80**	0.86	0.57	1.30	**1.23**	**1.08**	**1.41**
Age 35–49	**8.14**	**4.94**	**13.40**	**8.32**	**5.26**	**13.17**	**2.47**	**1.71**	**3.57**	**2.19**	**1.93**	**2.49**
Age 50–64	**20.82**	**12.70**	**34.13**	**12.96**	**8.21**	**20.46**	**4.25**	**2.96**	**6.09**	**2.93**	**2.58**	**3.33**
Age 65–79	**45.05**	**27.50**	**73.83**	**14.63**	**9.26**	**23.13**	**6.11**	**4.26**	**8.76**	**3.83**	**3.37**	**4.36**
Age ≥ 80	**82.88**	**50.53**	**135.95**	**6.75**	**4.23**	**10.77**	**4.35**	**3.01**	**6.28**	**3.60**	**3.15**	**4.11**
Comorbidity^¥^												
CCI 1–4	**3.06**	**2.86**	**3.28**	**3.68**	**3.38**	**4.01**	**4.60**	**4.10**	**5.16**	**3.36**	**3.21**	**3.52**
CCI ≥ 5	**6.31**	**5.83**	**6.83**	**5.00**	**4.49**	**5.57**	**9.13**	**8.02**	**10.39**	**6.80**	**6.41**	**7.22**
Hospital size^ψ(bed number)^												
Medium (300–499)	**1.39**	**1.16**	**1.65**	0.87	0.70	1.08	0.91	0.73	1.14	**1.41**	**1.23**	**1.63**
Large (500–999)	**1.67**	**1.43**	**1.95**	**1.25**	**1.05**	**1.49**	**0.68**	**0.56**	**0.83**	**1.79**	**1.58**	**2.03**
Very large (≥ 1000)	**1.25**	**1.07**	**1.45**	1.04	0.88	1.22	**0.70**	**0.58**	**0.84**	**2.11**	**1.87**	**2.37**
US region^§^												
Northeast	**1.30**	**1.22**	**1.39**	1.09	0.99	1.20	**1.87**	**1.69**	**2.06**	**1.51**	**1.43**	**1.58**
Midwest	1.00	0.91	1.11	**1.91**	**1.04**	**1.37**	1.00	0.85	1.18	**0.61**	**0.56**	**0.67**
West	**1.25**	**1.18**	**1.34**	**1.99**	**1.83**	**2.16**	**1.53**	**1.39**	**1.68**	**1.54**	**1.47**	**1.61**
% Concordant	84.6			77.1			80.1			77.1		

Reference Comparison *White Race, **Female Sex, ***Age < 18, ^¥^CCI = 0, ^ψ^Hospital Bed Size < 300, ^§^US South. Bold text confers statistical significance

Patients aged 50–64 and 65–79 years accounted for the largest proportion of all outcomes studied except mortality. Patients ages 65–79 had the highest case rates of ARDS and mechanical ventilation of 7.92% (p < 0.00001) and 6.98% (p < 0.00001), respectively. This was significantly higher than the younger groups (Table 2). On multivariate analysis, age was an independent risk factor for ARDS with increasing odds risk for every age group until age 80. Increasing age was also a risk factor for mechanical ventilation above 35 years of age, rising with each age group until age 80. Those above 80 years had slightly improved risk of mechanical ventilation (OR 4.35, CI 3.01–6.28) from the 65–79 group (OR 6.109, CI 4.26–8.76) (Table 3).

Sepsis developed in 16,145 patients, of whom 16.2% were > 80 years old, 5068 (31.4%) were 65–79, 4590 (28.4%) were 50–64, 2454 (15.2%) were 35–49, and the remainder were in those < 34-year-old. Case sepsis rates increased with every age group age until age 65. The groups 65–79 years and > 80 years were numerically similar at 27.67% and 28.95% (p = 0.0271), respectively. Multivariate analysis identified age as an independent risk factor for sepsis- increasing odds with increasing age (Table 3).

### Impact of gender

Nearly half of the 100,902 total cohort were females (50.7%) showing equal prevalence of COVID-19 in women. Of the 8574 patients who died, 58.2% were male and 41.3% were female. The case fatality rate was 10.11% for males and 6.93% for females (p < 0.00001). On multivariate analysis, male gender was shown to be an independent risk factor for mortality (OR 1.49, CI 1.42–1.57) (Tables 2, 3).

Males were also found to have increased risk of ARDS (OR 1.509, CI 1.41–1.61) and sepsis (OR 1.38, CI 1.33–1.43). The case ARDS rate was 5.08% (2504) for males and 3.15% for females (p < 0.00001). The case sepsis rate was 18.52% for males and 13.54% for females (p < 0.00001). Gender was not found to be an independent risk factor for mechanical ventilation, with similar case rates for males 3.36% and females 2.94% (p = 0.0001) (Table 3).

### Impact of race

The majority of the study cohort was White (58.1%), with 18.4% identifying as Black and 23.5% as “other”. White patients made up an even higher proportion (62.8%) of the 8574 patients who died with a case fatality rate of 9.18%. While 17.3% of deceased patients were Black and 20% (1712) were other races, the case fatality rates were 7.98% and 7.22% respectively (p < 0.00001). In multivariate analysis, Black race was not found to be a risk factor for mortality while non-Black, non-White races did have a small but statistically higher risk of death compared to Whites (OR 1.101, CI 1.032–1.174) (Table 3).

On multivariate analysis, Black race had a decreased risk of mechanical ventilation or sepsis compared to White race (OR 0.75, CI 0.68–0.84) and (OR 0.90, CI 0.86–0.95) respectively. All “other” races were found to have an increased risk of ARDS (OR 1.44, CI 1.33–1.55) and death (OR 1.10, CI 1.03–1.17) (Table 3).

### Impact of multimorbidity

Nearly half (4711, 54.9%) of the deceased patients had a CCI of 1–4, corresponding to an estimated 10-year survival of 53–96% [11]. Of the 36,072 admitted patients with CCI of 1–4, the case fatality rate was 13.06% compared to 29.6% (p < 0.00001) for patients with CCI ≥ 5 (estimated 10-year survival ≤ 21%) [11] who made up only 8.9% of the cohort. The case fatality rate for patients with CCI = 0 was 2.16%. Multivariate analysis showed a 3 and sixfold increase in odds risk for death for CCI 1–4 (OR 3.06, CI 2.59–3.28) to CCI ≥ 5 (OR 6.31, CI 5.83–6.83) (Table 3).

Patients with CCI ≥ 5 had a case ARDS rate of 9.65%, case ventilation rate of 10.79% and case sepsis rate of 42.75%. Those with CCI of 1–4 had a case ARDS rate of 6.84%, case ventilation rate of 5.04%, and case sepsis rate of 24.34%. Those with CCI of zero had a case ARDS rate of 1.43%, case ventilation rate of 0.7% (391) and case sepsis rate of 6.31% (3,526). On multivariate analysis, increasing CCI score was consistently an independent risk factor for all poor outcomes (Table 2).

### Hospital characteristics and impact on morbidity and mortality

The largest subgroup of patients in our cohort were from hospitals in the Southern region of the United States (40.1%) followed by the West (31%), the Northeast (20.6%), and the Midwest (8.2%). Of the deceased patients, the South had the highest proportion with 35.7%, followed by the West with 30.9%, the Northeast with 25.7%, and the Midwest with 7.6%. Despite these proportions, the South had the lowest case fatality rate of 7.57%, followed by the West with 8.47%, and finally the Northeast at 10.58% (p < 0.00001). The Midwest made up the smallest portion of admissions and deaths in the cohort, yet the regional case fatality rate was higher than the South at 7.87% (p = 0.352) although insignificant. Multivariate analysis validated a higher independent risk of mortality for the Northeast (OR 1.299, CI 1.22–1.29) and West (OR 1.26, CI 1.18–1.34) (Table 3).

Very large hospitals (> 1000 beds) accounted for 66.7% of overall admissions and 65.9% of all deaths. Large hospitals (500–999 beds) accounted for 25.3% of deaths, medium hospitals (300–499 beds) for 6.3%, and just over 100 deaths occurred at hospitals with 200–299 beds and < 200 beds. The case fatality rate was highest for large hospitals (500–999 beds) at 10.35% followed by hospitals with > 1000 beds at 8.39% and 6.76% for medium sized hospitals (p < 0.00001). In multivariate analysis, larger hospitals had an increased risk of mortality, highest at medium sized hospitals (OR 1.67, CI 1.43–1.95) (Table 3).

## Discussion

To our knowledge, this study represents the largest cohort of hospitalized patients with COVID-19 in the United States from December 2019 to November 2020. Advanced age, male gender, and greater CCI were identified as independent risk factors for poor outcomes in hospitalized COVID-19 patients across the US. Our study shows worse outcomes in the Northeast and West regions of the United States for those hospitalized with COVID-19 during this time period. No significant differences in severe outcomes including ARDS, sepsis and death were found between Blacks and Whites, but worse outcomes were noted in those of other minority groups.

We found an overall case fatality rate of 8.5% in the hospitalized population. Males had a higher rate of all poor outcomes and increased risk of death compared to women which has been shown in prior studies [12, 13]. Our study also validates previous findings of a strong link between increased age and CCI with poor outcomes [1, 3, 13, 14]. Age had the largest influence on risk of death whereas the CCI had the largest impact on predicting need for mechanical ventilation. Studies have found some comorbidities, namely hypertension, diabetes and cardiovascular disease to be common in patients with COVID-19 infection, particularly severe infection [2–4, 8, 15] compared to those who do not contract COVID-19. In keeping with this trend, the most common comorbidities in our cohort were hypertension and diabetes.

We also found a significantly higher risk for mortality, mechanical ventilation and sepsis for hospitals in the Northeast and West regions of the United States. The higher case rates for fatality and severe complications in the Northeast and West regions can be explained by the natural history of disease surges experienced in the United States, first in the Northeast and then in California and Seattle [16]. Yancey et al. made the clever observation that social distancing, a key practice known to decrease the risk of infection, is a privilege [17]. This social reality can explain the compounding effects that underlying social disparities are having on multiple skewed trends for COVID-19 infection, hospitalization, and worse outcomes. The Northeast and West regions of the United States have higher population densities compared to other regions, predominantly in urban settings [18, 19], making social distancing practices more difficult at times. Case fatality rates were higher at larger hospitals as well. This can be attributed to the notion that patients with more severe infection were transferred to referral centers with higher level of intensive care which is not always available at smaller institutions.

The regional trends of infection highlighted here and concept of population disparities brings forth the extremely important discussion of race and COVID-19. During the first surge of the pandemic, rates of infection, hospitalization and worse outcomes in Black and other minority populations were alarmingly high [20–22]. After correcting for risk factors including comorbidities and age, early studies found higher rates of infection and intensive care needs in minority, non-White populations [5, 6, 14, 23, 24]. However, most of these studies were single center cohorts, in urban settings or were from early in the pandemic when crowding and high exposure was prevalent. Our study found in nearly 100,000 admitted patients with COVID-19, during similar time course, with regional distributions representative of the general US population, show no increased risk of death between Blacks and Whites. Other studies with greater longitudinal data have shown there to be an increased risk of mortality for Blacks, however our study did not [5, 7, 23]. This suggests, while infection rates are higher in minority populations, once admitted to a hospital with appropriate treatment, this group may not be at higher risk of death. It is unclear how the care of different races at hospitals using Cerner EHR may differ from that of prior studies. Our study did find that other races (including Asians or those who identify as “other” race) have an increased risk of death and ARDS, however. Nau et al. noted a significant increased risk for hospitalization and intensive care needs for Pacific Islanders compared to Whites [6]. These observations deserve further evaluation. Our data supports the assertion that race can increase ones risk for acquiring COVID-19 and severe complications [25–27]. These groups often are impacted by social disparities, such as living in crowded, multi-family homes areas, less access to healthcare and variable insurances are examples of systemic racism that requires dismantling.

Despite the strengths of our large population-based cohort, our study has limitations. First, being a retrospective database study, our data is limited to that which was coded during patients’ hospitalizations and subject to bias by those documenting and coding encounters. The individual clinical picture is not known. The exact clinical reason for admission is not identifiable and patient visits were tied to their primary diagnosis only. The data was limited to the first encounter associated with COVID-19, likely missing outcomes later in the disease course of some patients. Our study represents hospitalized patients with COVID-19 infection only. This skews data from those with milder infection or those seeking ambulatory care. Finally, the use of the Cerner database may be self-selective of a particular patient population. This study population may exclude smaller facilities, safety net hospitals, and particularly, the Veteran population through the VA system which use different EHRs.

## Conclusion

To close, our large, nationwide study shows age and high comorbidity scores are independent risk factors for poor outcomes during hospitalization for COIVD-19. With respect to race, Black race was not an independent risk factor for worse outcomes compared to Whites in this cohort. However, the trend for increased hospitalization and infection in Black individuals for COVID-19 have been shown in multiple studies and warrant discussions regarding health care disparities in different hospital systems in the United States. We do show an increased risk of mortality and ARDS in other minority groups. Again, this highlights more groups deserving increased attention, and need for investigation as to how this systemic racism can be mitigated.

## Supplementary Information

**Additional file 1.** **Supplementary Table 1.** ICD-9, ICD-10 and qualifying diagnosis and lab codes used for data requisition in CRWD database.

## Data Availability

The data used for this study is from the CRWD COVID-19 Database and is available upon request by emailing: learninghealthnetwork@cerner.com or visiting https://www.cerner.com/solutions/real-world-data. The database was open access at the time of collection, and public access can be sought by contacting the links above.

## Abbreviations

CRWD: Cerner Real World Data COVID-19 database
EHR: Electronic health record
COVID19: Infection and disease caused by SARS-CoV-2 virus
SARS-CoV-2: Severe acute respiratory syndrome corona virus 2
ARDS: Acute respiratory distress syndrome
OR: Odds ratio
CCI: Charlson Comorbidity Index
