# Supplementary material for: Impact of age, sex, race, and regionality on major clinical outcomes of COVID-19 in hospitalized patients in the United States
Source: BMC Infect Dis. 2022 Jul 29;22:659. doi: 10.1186/s12879-022-07611-z (PMC9335459; doi:10.1186/s12879-022-07611-z)
Supplement: Supplementary file 1 — Additional file 1. Supplementary Table 1. ICD-9, ICD-10 and qualifying diagnosis and lab codes used for data requisition in CRWD database. [file 12879_2022_7611_MOESM1_ESM.docx]

Supplementary Table 1. ICD-9 (ICD-10) codes used for data requisition

| Code | Code Description |
| --- | --- |
| K92.0, K92.1, K92.2 (578.0, 578.1, 578.9) | Gastrointestinal hemorrhage (GI bleed) |
| N17% (584%) | Acute kidney injury |
| J80 (518.82) | Acute respiratory distress syndrome (ARDS) |
| Z99% (V46%, V45.1%) | Dependence on ANY enabling machines and devices (dialysis, oxygen supplement of any kind) |
| Z99.1% (V46.1%), 40617009 | Dependence on respirator (ventilator) status ONLY |
| A41%, R65.1%, R65.2% (995.9%, 785.5%) | Sepsis (with or without specified organism) |
| K56.6%, K56.7%, K59.8% (560.8%, 560.9, 564.89) | Intestinal obstruction |
| R74.0, R94.5 (790.4, 794.8) | Transaminitis |
| A04.7 (008.45) | C. diff |
| I82.5%, I82.7% (453.5%, 453.7%); I82.8% (453.6%) | CHRONIC VTE (deep and superficial); unspecified time |
| I82.4%, I82.6% (452.4%, 453.8%) | ACUTE VTE (superficial and deep) |
| J44%, J45%,J98.2, J98.3 (493%, 496, 518.1%, 518.2%) | COPD, Asthma, Emphysema |
| J84.1%, J84.9 (515%, 516.3%, 516.9%) | Pulmonary fibrosis |
| E08%, E09%, E10%, E11%, E13% (249%, 250%) | Diabetes |
| I10, I15% (401%, 405%) | HTN |
| I12.0, I13.1%, I13.2%, N18.6 (404.0%, 404.1%, 404.9%, 585.6) | End stage renal disease (ESRD) |
| M06.0%, M06.2%, M06.3%, M06.8%, M06.9%, M05.1%, M05.2%, M05.3%, M05.4%, M05.5%, M05.6%, M05.7%, M05.8%, M05.9% (7.14%) | Rheumatoid disease |

Legend: % denotes inclusion of all codes within whole value (i.e any codes with decimal values following the whole integer code)
